# Light People: Professor Yang Gao

**DOI:** 10.1038/s41377-022-00869-7

**Published:** 2022-06-27

**Authors:** Hui Wang

**Affiliations:** Changchun Institute of Optics, Fine Mechanics and Physics, Chinese Academy of Sciences, 3888 Dong Nan Hu Road, 130033 Changchun, China

**Keywords:** Photonic devices, Other photonics

## Abstract

The safe return to earth of the Shenzhou-13 crew not only marks a perfect ending to a successful mission, but also reignited the public’s interest in the exploration of space. This month’s Light People features a true “Space Explorer”. In the past two decades, she has devoted to space robotics research and is recognized for advancing AI capabilities for future space missions. In addition, she has been actively promoting STEM through public outreach and setting role models to encourage women in STEM education and careers. She is Professor Yang Gao, winner of the Mulan Award 2019 in science and technology, a dedicated award to celebrate the achievements and success of Chinese women in the United Kingdom and wider. Prof. Gao is the Professor of Space Autonomous Systems at the University of Surrey’s Space Centre, and founding head of the STAR LAB which specializes in robotic sensing, perception, visual GNC, and biomimetic mechanisms for industrial applications in extreme environments. She is also Editor-in-Chief of the Journal of Field Robotics, where she demonstrates leadership in serving the wider scientific community, strengthening academia-industry connections, and cultivating new generations of sci-tech talents. So what is Prof. Gao’s story with *Light*? What unique insights does she have on the subject of scientific research? Please sit back, fasten up your seatbelt, and follow Prof. Gao and Science Editor on a journey to outer space.

## Abstract (web summary)

Prof. Yang Gao, a true “Space Explorer”, has more than 20 years of experience in AI Robotics research and development for extreme environments such as space.

**Figure:**
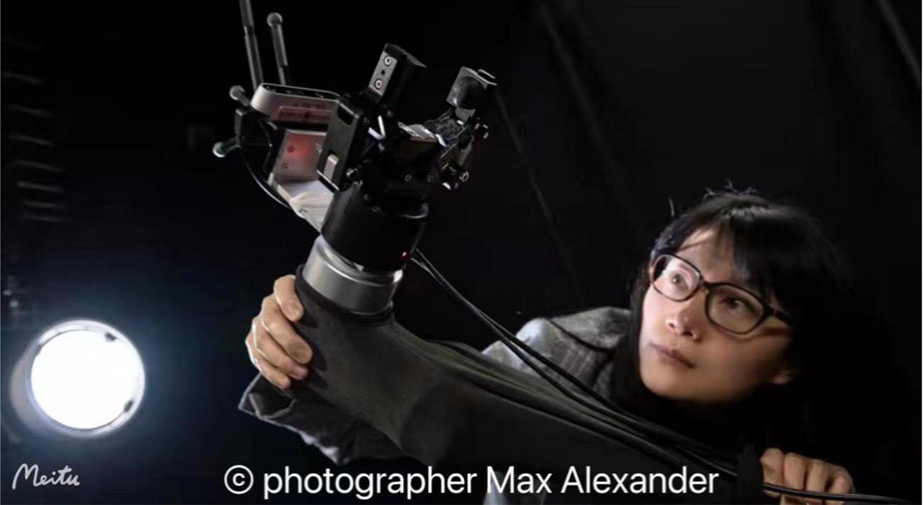

**Biography:** Professor Yang Gao, from the University of Surrey, UK, is a world-renowned expert in space robotics and the editor-in-chief of the *Journal of Field Robotics*, a top robotics journal. She has more than 20 years of experience in artificial intelligence research and development in extreme environments such as space. She is a Fellow of the Institute of Engineering and Technology (IET) and a Fellow of the Royal Aeronautical Society (RAeS). In 2008, she was named by Times Higher Education as one of the UK’s top ten outstanding young scholars who have made significant contributions to her discipline, and was awarded the Mulan Award in 2019 for her contributions to science, technology, and engineering.

She has served as a Principal Investigator (PI) of projects funded by the European Space Agency (ESA), the UK Space Agency, the European Commission, the UK Research Innovation, the Royal Academy of Engineering, as well as industry through international and national collaborations. Professor Gao is actively involved in real-world space mission design and development, to name a few, ESA’s ExoMars, Proba3 and VMMO, UK’s MoonLITE/MoonRaker, and CNSA’s Chang’E 3.

Professor Gao’s research team has received many international recognitions or awards, such as the 2013 IAF Edmund Bren Silver Medal, the 2016 COSPAR Outstanding Paper, the 2018 ESA SysNova Challenge Joint Champion, the 2019 IEEE/ASME-AIM Best Paper Finalist, the 2019 European Space Agency-Stanford Challenge Top 3, and the 2020 IEEE-ICRA Space Symposium First Prize of Wiley Poster Award, etc.

**1. As a world-renowned expert in space robotics, can you briefly introduce your research direction and content?**

I specialize in R&D in robotic sensing, perception, machine learning, visual navigation, and bionic mechanisms, as well as their applications in the extreme environments for sectors like the space, nuclear, and utility. I have international research projects funded by the European Space Agency, UK Space Agency, European Commission, UK Research Innovation, Royal Academy of Engineering, and various industrial companies. I have also been involved in real-life space mission development, such as ESA’s ExoMars, Proba3 and VMMO, UK’s MoonLITE/MoonRaker, or collaborating with the Chinese Academy of Sciences on deep data analysis collected by the Chang’E 3 lunar rover.

**2. What is the current focus of your research? What problems are you working on?**

My current research focus on addressing challenges in spacecraft autonomy in-orbit or extraterrestrial bodies where the spacecraft are required to navigate and operate autonomously.

For orbital robots, such as those used for in-orbit servicing or removal of space debris, need to be immune to sensor noise and uncertainties in space to achieve autonomous navigation capabilities. There are further challenges due to limitations of the spacecraft hardware since the onboard computer needs to be radiation hardened. Therefore, the computational speed is relatively slow, which requires the intelligent algorithm of autonomous navigation to be low in computational complexity. To tackle these technical challenges, we have developed intelligent algorithms for spatial navigation using only monocular camera sensors, and methods for robotic hand grasping of cooperative/non-cooperative objects based on sparse point cloud sensing.

For planetary robots such as for Mars, the robots need to deal with varying surface features on Mars, from rugged terrain to loose sand. There are also interesting features inside the Martian regolith that requires drilling and sampling to study. To address these needs, we have developed a planetary sampling rover using principle of bionics. The rover can perform complex but energy-efficient locomotion such as crawling, climbing, and burrowing. The drilling technique is inspired by wasp ovipositor hence the robotic prototype is developed in low mass and size hence suitable to achieve low-energy drilling and sampling in low gravity, such as Moon, Mars, and asteroids.

**3. The United Nations Office for Outer Space Affairs (UNOOSA) has appointed you as a Class of 2021 mentor for the Space4Women project, which aims to promote active and equal roles for women in space science and technology, innovation and exploration, why did you participate in this initiative? How do you feel about it?**

I am passionate and committed in supporting and mentoring female students and young researchers through universities and different foundations, and aim to contribute my mentoring experience to the Space4Women program too. As a Space4Women mentor, I act as an ambassador to help implement the vision of the Space4Women by actively encouraging and empowering women in education and careers of Science, Technology, Engineering, and Mathematics (STEM). In addition, I carry out public engagement and outreach activities to promote Space4Women through a series of school visits, presentations, media interviews, science fairs, and festivals.

**4. You are the editor-in-chief of the** ***Journal of Field Robotics*, a top journal in the field of robotics. What are the main features of this journal? What are your experiences and feelings as editor-in-chief?**

*Journal of Field Robotics* (JFR) is an international academic publication focusing on “Field Robotics”. It has a history of more than 15 years and is recognized as a Q1 journal in computer and engineering-related disciplines. Field robots mainly refer to robots working in unstructured and dynamic environments, involving a wide range of industrial applications, including construction, forestry, agriculture, mining, nuclear energy, submarine, smart highway, search and rescue, military, and aerospace. JFR publishes papers with theoretical foundation and practical validation. We encourage authors to clearly articulate the theoretical rationale of the scientific claims and demonstrate real-world applications with experimental data and validation. We aim to report not only the authors’ scientific work, but also the lessons learnt gained from practical field trials.

As the Editor-in-Chief of JFR, my goal is to serve all the relevant scientific and technological communities over the globe. Our editorial team is committed to discovering and publishing high-quality academic results and papers in a timely manner through a rigorous peer-review system. We actively maintain contacts with relevant academia and industry to influence and help promote rapid development of the field of robotics, which also includes helping to promote Diversity, Equality, and Inclusion in related research fields and industries (such as promoting gender equality).

**5. You have taken part in the Light Conference hosted by** ***Light: Science & Applications*** **(Light), what was your experience? What advice do you have for** ***Light*?**

I had the pleasure to have participated in the Light Conference organized by *Light: Science & Applications* as a conference co-chair. I was very impressed by the sessions I attended. The conference featured keynotes by international leading experts and facilitated technical discussions within the community.

On the 10^th^ anniversary of the publication of *Light*, I wish the journal continues to maintain a high level of academic standard and provide publication opportunities and communication vehicle for the relevant scientific and industrial community. As an internationally renowned journal, *Light* can serve as a good platform to help facilitate exchanges of scientists and industrialists in the sector, as well as the wider international cooperation.

**Figure:**
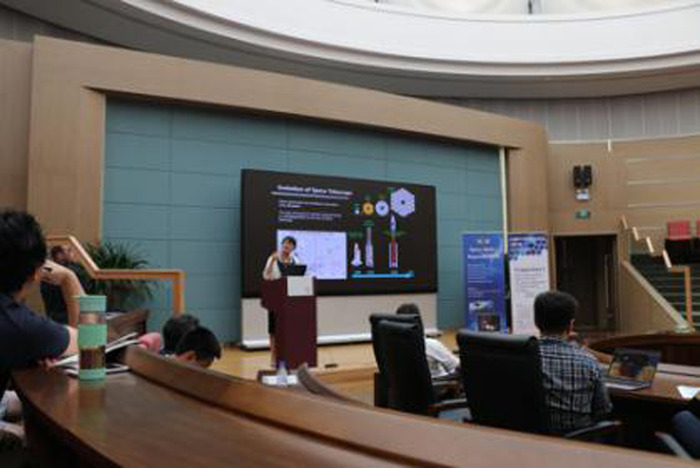
Prof. Yang Gao made a presentation at Light Conference 2018

**6. You are the Founding Director of the Autonomous Robotics Laboratory (STAR LAB) at the Surrey Space Centre, University of Surrey, UK. What difficulties did you encounter in the early days of the lab?**

Challenges on creating and leading a research lab are ongoing issues facing its leaders. In the start-up period, the main challenge is to establish scientific priorities and to develop our unique research identity or brand by striving for innovation and outstanding scientific insights. Following from there, the challenge is around consolidating research directions and scientific philosophies or approaches to help produce theoretical findings and practical verification results. It is also very challenging to continue to grow/sustain the team, attract/cultivate next generations of potential researchers, and maintain high-quality/quantity outputs.

**7. You won the first prize of the** **14**^**th**^ **“China-UK Entrepreneurship Competition”. How do you view the relationship between scientific research and industry?**

Scientific research and industrialization represent different stages of maturity in science and technology. The scientific research stage usually refers to Technological Readiness Level (TRL) 1 to 4, which basically refers to the stage of exploration and validation of new knowledge. Industrialization refers to TRL 5 to 9, relating to the stage of more in-depth development of technology and commercialization of products. Traditionally, the research carried out at the university campuses corresponds to low TRL. With the booming of high-tech and deep-tech sectors, many university-oriented research work becomes more suitable for commercialization and industrialization. This is one of the reasons why we participated in the Entrepreneurship Competition and won the award given our expertise.

**8. As a doctoral supervisor, you have trained many students. What abilities do you most want your students to develop? What do you expect from the students?**

In PhD supervision, I pay great attention in help developing students’ scientific rigor and integrity. It is very important to possess an honest and respectful attitude towards science. In the journey of scientific research, one should aim to be rigorous, creative, open-minded, resourceful and supportive to peers, grateful, and responsible to the society.

**9. You once said, “When I was a kid, I was very into engineering. I like to do things myself. I was curious about all things machine.” How did that passion for machines and engineering affect your career choice?**

My childhood interests or innate love for technology had a huge impact on my career choice and continued development. This allows me to choose a career of dream and interests. Especially when encountering difficulties, I often feel more empowered and confident to overcome obstacles, and rarely spend time complaining, self-pity, or put yourself in a negative state of mind.

**10. You have won many international awards. In 2019, you were awarded the “Mulan Award” for your contributions to science, technology, and engineering. What do these awards mean to you?**

It is an honor and a privilege to be recognized by an important and essential organization as the Mulan Foundation. I feel very strongly that women of all nationalities play a critical role in helping to progress and improve their professions, their communities, and the world and we must do all we can to recognize and lift them up.

**Figure:**
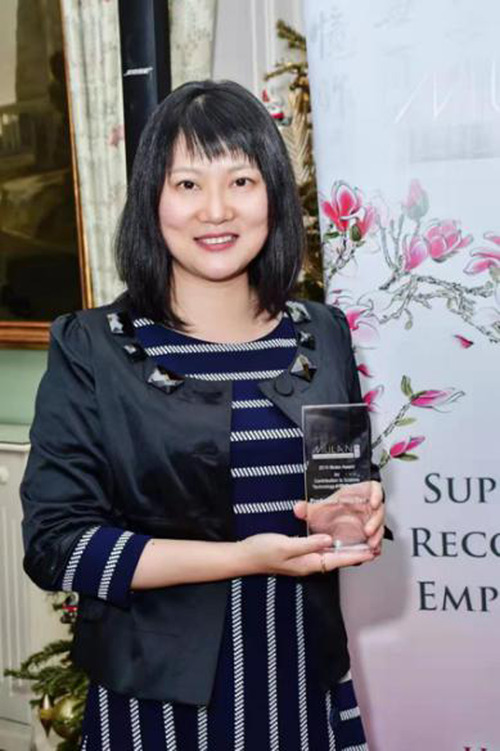
Prof. Yang Gao was awarded the “Mulan Award”

**11. On International Women’s Day in 2021, you participated in an interview with Wiley and sent holiday wishes. What do you think are the advantages of women in scientific research?**

I am a firm believer in the principles of “Equality, Diversity, Inclusion (EDI)” and the importance of EDI in the field of research. Everyone deserves equal opportunities to pursuit scientific research. People regardless of gender can potentially play important roles when they demonstrate required scientific abilities, commitments, and sense of responsibilities. I think women are the same as men in front of science, and neither side has unique advantages over the other.

**12. Can you name one important turning point in your life please?**

Take career as an example, shifting my research focus to space robotic applications is an importance turning point. This happened nearly 20 years ago by chance, when project opportunities arose from the space industry to investigate AI robotics for future space applications. I took the opportunity and made a step change in my career direction and priority and never looked back since then.

**13. Name one scientist who you admire the most, and tell us why?**

My role models are two Nobel Prize winning female scientists, Marie Curie and TU Youyou. I am inspired by their determination and work ethics in pursuing scientific research, despite the limited resources and poor working conditions they had to work around at their time.

**14. What hobbies do you have?**

In my spare time, I like to explore and learning new things too, from self-taught piano playing, handcraft making, to studying architecture, landscaping, and many more.

**15. What qualities do you think an excellent scientific researcher should possess?**

For scientists, academic integrity, rigor, and freedom are very important. These qualities help retain the resilience and independence of academia to help enable the scientific community conduct research responsibly and apply knowledge for sustainable development of the society.

## Light special correspondent

*Hui Wang is the Deputy Director of the Office of International Cooperation in the Changchun Institute of Optics, Fine Mechanics and Physics (CIOMP), Chinese Academy of Sciences (CAS). She currently works on international communication and cooperation for the CIOMP and was a founding member for the Nature Publishing Group and CIOMP joint journal Light: Science & Applications. She is the founder of “Rose in Science” and has published several articles in Acta Editologica, International Talent, Light: Science & Applications, etc., and was invited to take an interview by SPIE Women in Optics, which was published in 2015.*

